# Analysis of salt-bridges in prolyl oligopeptidase from Pyrococcus furiosus and Homo sapiens

**DOI:** 10.6026/97320630015214

**Published:** 2019-03-15

**Authors:** Amal Kumar Bandyopadhyay, Rifat Nawaz Ul Islam, Debanjan Mitra, Sahini Banerjee, Arunava Goswami

**Affiliations:** 1 Department of Biotechnology, The University of Burdwan, Burdwan, West Bengal, India; 2 Department of Zoology, The University of Burdwan, Burdwan, West Bengal, India; 3 Department of Biological Sciences, ISI, Kolkata, West Bengal, India

**Keywords:** Thermo stability, salt bridge, salt bridge design, electrostatics, prolyl oligo peptidase

## Abstract

Hyper thermophilic archaea not only tolerate high temperature but also operate its biochemical machineries, normally under these conditions. However, the structural signatures in proteins that answer for the hyper thermo-stability relative to its mesophilic homologue remains poorly understood. We present comparative analyses of sequences, structures and salt-bridges of prolyl-oligopeptidase from Pyrococcus furiosus (pfPOP - PDB ID: 5T88) and human (huPOP - PDB ID: 3DDU). A similar level of hydrophobic and hydrophilic residues in pfPOP and huPOP is observed. A low level of interactions between shell-waters and atom-types in pfPOP indicated hyper thermophilic features are negligible. Salt-bridge-forming-residues (sbfrs) are high in pfPOP's core and surface (pfPOP). Increased sbfrs largely indicate specific-electrostatic is important for thermo stability in pfPOP. Four classes of sbfrs are found namely positionally non-conservative (PNCS), conservative (PCS), unchanged (PU) and interchanged (PIC) type of substitutions. PNCS-sbfrs constitutes 28% and it is associated with the topology of pfPOP at high temperature. PCS helps to increase the salt-bridge population. It is also found that PU maintains similar salt-bridges at the active site and other binding sites while PIC abolishes mesophilic patterns.

## Background

Native state of the protein is thermodynamically compromised. Thus, both favorable and unfavorable weak interactions are involved in this state of which salt bridge has been a major contributor to the thermostability [1]. A salt bridge is defined as a specific electrostatic interaction between the side-chain of basic and acidic amino acids in native protein [2, 3]. Since the tertiary structure of protein is formed by the stabilizing contribution of weak interactions and since these forces show wide modulation, researches have been conducted to investigate dominant force in the folded state of proteins operating under extreme of environmental conditions. Computation of energetics of salt bridge has made phenomenal progress in recent time [4, 5]. Proteins functioning under the extreme of temperature, high-salt, and other hostile conditions show strict dependence on these environmental parameters for stability and functionality [1, 6, 7, 8]. Comparative studies involving various physicochemical and structural parameters such as amino acids composition, oligomeric state, hydrophobicity, compactness, helical-content, salt-bridges, buried and exposed surface area and etc for homologous pairs of thermophilic and mesophilic protein showed insight into thermophilic adaptation [9] of which the increase of ion-pairs in thermophilic proteins have been somewhat general [10]. It has also been observed that the stability of thermophilic proteins is directly correlated with the specific electrostatic interactions [11]. Using glutamate dehydrogenases from the thermophilic and mesophilic organisms, it has been demonstrated that not only the frequency but also the stability of salt bridge is greater in the former [1] than the latter. The stabilizing and destabilizing roles of salt-bridges are largely dependent on their location either in the protein's core or surface. By computational and experimental studies, it has been demonstrated that the buried salt-bridges are more stabilizing than the exposed ones [1, 2, 12]. Oppositely, it has been claimed that buried salt-bridges fail to overcome the desolation cost and thus are mostly destabilizing [13]. Alternately, if salt-bridge forming residues are substituted by hydrophobic isosteres, stability increases [13]. This conjecture is supported by a designed experiment that by removing a salt-bridge triad (networked) from the core of Arc-repressor by various combinations of hydrophobic isosteres not only increase the net stability by 2-4 kcal/mol than the wild-type protein, but also the specificity of mutant-variants remains similar as the wild-type protein [14].

Comparative analyses of the electrostatic contribution of orthologous thermophilic and mesophilic proteins have been an important and active research area [15]. Our understanding of the stabilizing and destabilizing effects of buried and networked saltbridges still remains an enigma [2, 13, 14]. The dielectric constant under the hyperthermophilic condition is drastically low (~55), yet hydrophobic force was proposed to be the dominant contributor to thermostability. On the other hand, at high temperature, the solvation of charged amino acids is severely affected, which would facilitate easy desolvation of the partners of salt-bridge and thereby making the latter stabilizing [1]. The role of specific-electrostatic interactions in hyperthermophilic archaea, Pyrococcus furiosus and human prolyl oligopeptidase (pfPOP and huPOP) are yet to be understood. Prolyl endo peptidase is a serine protease that cleaves peptide at internal Proline sides. While catalytic triad forming residues, SER, HIS, and ASP are similar as other serine proteases (trypsin, subtilisin etc), the overall structure of the enzyme is different [16]. POP's molar mass is about 3 times higher than other proteases. Relative to huPOP, pfPOP has a temperature optimum of 85(°C). At this temperature (<85°C), pfPOP is stable for 12 hours [17]. pfPOP is shorter by 94 residues compare to huPOP [18]. The structures of pfPOP and huPOP are solved at 1.9 Å and 1.56 Å resolutions respectively. The protein has two domains, the catalytic domain, and the β-propeller domain. The catalytic domain, which is constituted by two different sequence segments of POP, is present at the C - terminal end. The β-propeller domain is situated in between these two segments [19]. Structural information of pfPOP in the form of literature is not yet available, although the structure of the protein is solved at high resolution. In this study, we undergo extensive analysis of the salt-bridge pattern of pfPOP in comparison to its human homologue (huPOP). The study involves sequence, structure and evolutionary criteria along with detailed binary items of salt-bridges to gain insight into the structural features responsible for the thermophilic adaptation of pfPOP. The study also highlights the substitution pattern of salt-bridge forming residues in aligned sequence. This analysis highlights the evolutionary effects of thermophilic adaptation of pfPOP in comparison to its human homologue (huPOP). Overall, our study involves comparative analysis on salt-bridges, which we believe would have potential applications in protein-engineering and structural bioinformatics.

## Methodology

### Dataset:

The 3D structure of pfPOP and its homologous huPOP are procured from the Research Collaboratory for Structural Bioinformatics (RCSB) protein data bank (PDB) [20]. Sequence identity of pfPOP and huPOP are 29%, although both are functionally identical. Few important structural features that are procured from the RCSB summary pages are shown in Table 1.

### General comparative analysis:

Detailed analysis on physicochemical and sequence properties along with preparation of BLOCK of pfPOP and huPOP are done using PHYSICO [22] and PHYSICO2 [23]. The sequence BLOCK of pfPOP and huPOP are used for the analysis of evolutionary parameters using APBEST program [24]. Salt bridges are computed using SBION [25] and SBION2 [26] programs. Notably, although salt bridge analysis is possible using other analytical programs [27], residue-specific binary items could only be analyzed by SBION2. The core and surface compositions of crystal structures of POP are extracted using COSURIM [28].

A structural analysis of huPOP (3DDU) is performed on A-chain. In 5T88 (pfPOP), there are two chains (A and B). Chains are separated before any structure related analysis. The structure of huPOP and pfPOP are minimized for 1000 steps using AUTOMINv1.0, if not mentioned otherwise [29]. The shell-water interactions are analyzed on structures of huPOP and pfPOP using POWAINDv1.0 [30].

### Salt-bridge's binary items:

Extraction of salt-bridges is performed on un-minimized structures of huPOP (3DDU) and pfPOP (5T88). Salt-bridges thus obtained per-structure is divided into two categories: isolated and networked [31, 2]. Each of this binary category is then divided into two classes: core and surface [28]. Now, each of this four classes (namely: isolated-core, isolated-surface, networked-core, and networked-surface) are further grouped into single-bonded vs multiple-bonded, local vs non-local, salt-bridges in secondarystructure (helix and strand) vs salt-bridges in the coil, hydrogenbonded vs non-hydrogen-bonded categories. Some more subclasses are also made, such as salt-bridge in intra-helix vs saltbridges in intra-strand, salt-bridges in inter-helix vs salt-bridges in inter-strand. Although all these terms are extracted directly in an automated manner [26], there is certain qualitative checking of the protein prior binary item analysis, which is performed by its lower version [25].

### Alignment and homologous positional analysis of salt-bridge forming residues:

The sequence of huPOP (UniProt ID: P48147) and pfPOP (UniProt ID Q51714) are extracted from UniProt [32] database. The FASTA files are aligned using T-COFFEE program [33]. The alignment is then used manually to position salt-bridge forming residues, which are procured from the supplementary table of SBION2. The substitutions in pfPOP are divided into four classes based on the types of substitution. If the substitution is hydrophobic to hydrophilic it is taken as NCS (non conservative substitution) type (marked by blue shade). If the substitution is hydrophobic to hydrophobic or hydrophilic to hydrophilic it is CS (conservative substitution) type (marked by green shade). If the substitution is acidic to basis (relative to huPOP), it is taken as design-changer (marked by red color shade). Unchanged partners are shown by cyan-color shade.

## Results

### General characteristics of pfPOP and huPOP:

The enzyme prolyl oligopeptidase is a typical protease, which possesses the same catalytic triad, which is constituted by SER-HISASP residues. However, although it possesses the same catalytic residues, it has remarkable differences from other proteases such as trypsin, chymotrypsin and subtilisin [17]. The enzyme, which is an internal proline cutter, is quite abundant in human and hyperthermophilic organism, Pyrococcus furiosus. Unlike huPOP, pfPOP functions optimally at 85°C to 90°C in the cytoplasm [16]. Do these enzymes differ in sequence and structural properties? We are interested to identify the sequence and structural features and to correlate such differential with the gain in stability under hyper temperature conditions. To check these, we have degmade a detailed comparison of sequence and structure for these two proteins, whose results are presented in Table 2. Following points are noteworthy. First, the huPOP is longer than the pfPOP by about 100 residues. Alignment of two sequences showed 13 insertion regions. Second, although, length is shorter in pfPOP, its hydrophobic and hydrophilic composition in the sequence has been similar to huPOP. Third, aliphatic index of both these proteins is quite high (with little more in pfPOP). This parameter is the indicator of protein stability [34]. Forth, pI of pfPOP is lower than the huPOP indicating acidic residues are higher in the former. GRAVY shows that the hyperthermophilic pfPOP is more hydrophilic than huPOP. The NCS: CS is more in pfPOP than huPOP. A higher ratio indicates more incorporation of the non-conservation type of substitution (i.e. hydrophobic to hydrophilic and vice versa). Fifth, homologous positions of these two proteins show the remarkable difference (74.9%). Notably, such difference is not reflected in the overall compositions of these proteins (see above). How the class compositions (hydrophobic and hydrophilic residues) vary in the structures of these proteins? It is seen that in pfPOP, the surface is relatively less hydrophobic and more hydrophilic than huPOP. Surprisingly it is seen that cores of the protein possess a high amount of hydrophilic residues in both pfPOP and huPOP. In the case of pfPOP, acidic and basic residues are higher in the core and in the surface than that of huPOP. To check the contribution of shell-waters in the stability of these proteins, we made a detailed comparison between huPOP and pfPOP. Notable, the latter functions near the boiling point of water. It is noteworthy that the number of detected waters is much lower in pfPOP than huPOP. It is seen from the table that all type of interactions (at a distance =≤3.2Å) are much lower in pfPOP than huPOP. It is of interest as to how much of these interactions are happening in the core. Interactions in the interior of protein and cavity are the indicator of stability [35]. The latter fractions of shell-waters and protein interactions are also much lower in pfPOP.

### Binary characteristics of salt-bridges

**Figure 1:**
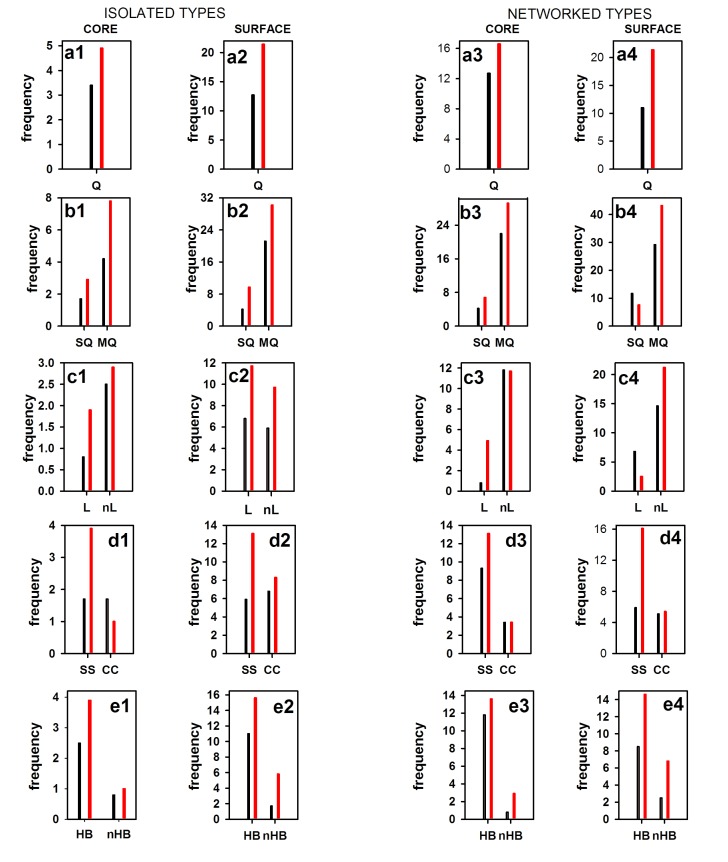
Category (Isolated vs Networked) and class-based (core vs surface) normalized frequencies of the binary item of salt-bridges from pfPOP (5T88_B; red) and huPOP (3DDU_A; black). Here sum of the binary items (e.g. HB and nHB) of any class is equal to the total frequency (Q) of that class.

To check the pattern of salt bridges for these two proteins (3DDU of huPOP and 5T88 of pfPOP), we have investigated the binary items, whose results are shown in Figure 1. Salt-bridges are divided into two categories i.e. isolated and networked type. Each of this category is then divided into two classes i.e. core and surface. For comparison purpose, the absolute frequency for each of this class is normalized as the length of huPOP and pfPOP are 710 and 616 respectively. Several points are noteworthy from the figure. First, the normalized frequency (Q) is higher in 5T88 than 3DDU in isolated-core (Figure 1, a1), isolated-surface (a2), networked-core (a3) and networked-surface (a4) cases. Similarly, for binary items such as single (SQ) vs multiple (MQ) bonded (Figure 1, b1-b4), local (L) vs non-Local (nL) (Figure 1, c1-c4), secondary-structured (SS) vs coiled-structure (CC) (Figure 1, d1-d4), hydrogen-bonded (HB) vs non-hydrogen (nHB) (Figure 1, e1-e4) salt-bridges are higher in 5T88 (pfPOP) than 3DDU (huPOP). Second, although 5T88 largely shows a higher proportion of binary items, there are few details here. In surface-networked case, SQ is less but MQ is more in hyperthermophilic 5T88 (pfPOP) (Figure 1, b4). In isolatedcore/ surface, networked-core/surface cases, secondary-structured salt-bridges are much higher in 5T88 than that in the coiled case (Figure 1, d1-d4). In some cases, the latter is lower in 5T88.

**Figure 2:**
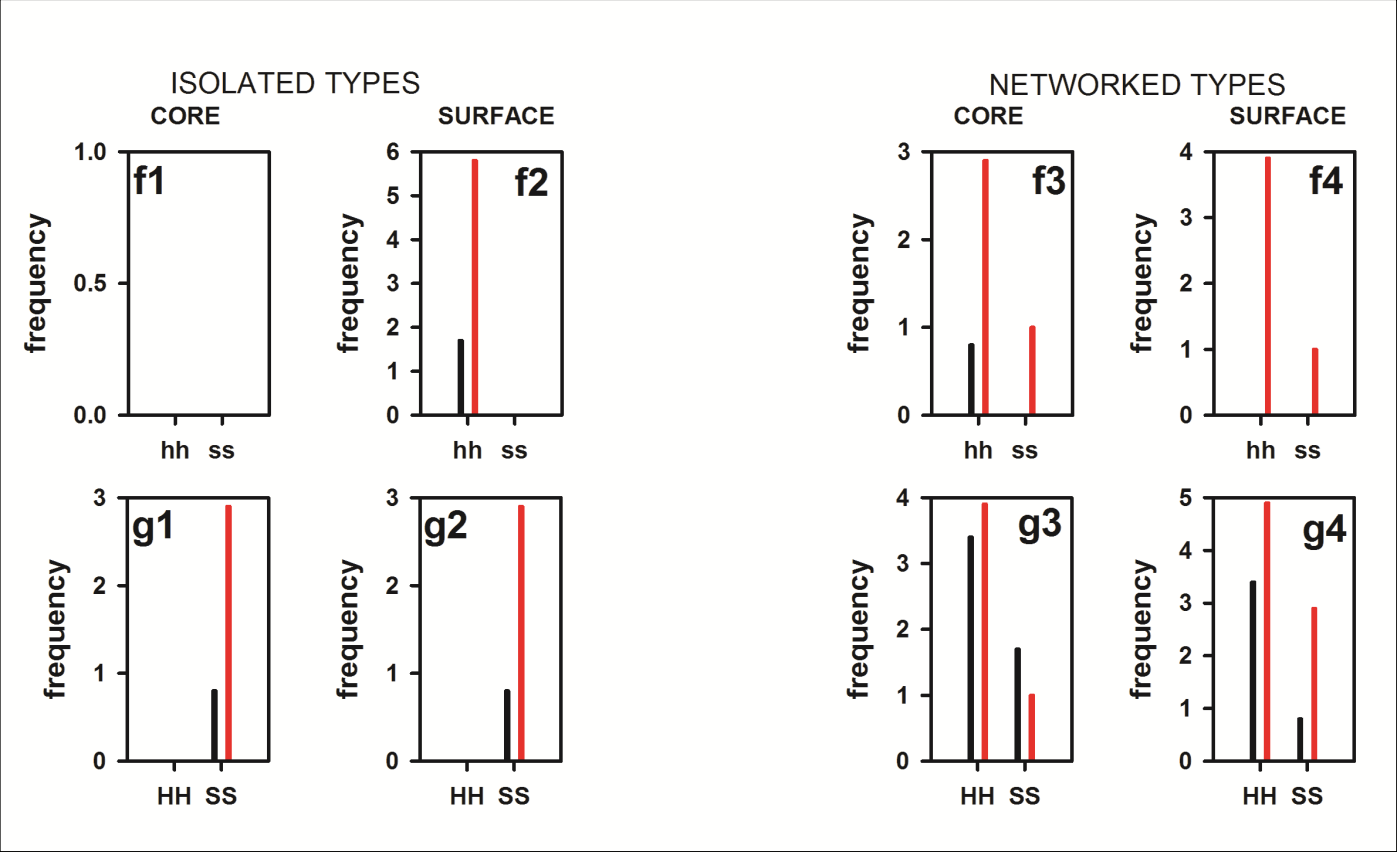
Partitioning of class-frequency of salt-bridges (isolated-core, isolated-surface; networked-core and networked-surfaced) into different secondary-structure combinations (hh, HH, HC, CH, HS, SH, ss, SS, SC, CS, CC), of which only hh (intra-HELIX), HH (inter- HELIX), ss (intra-STRAND) and inter-STRAND (SS) are shown. Red and black bars indicate the frequency of salt-bridges of 5T88 and 3DDU respectively.

There are nine combinations of secondary structures (S, H, and C), which are HH, HC, CH, SC, CS, SS, HS, SH, and CC. HH and SS can also be INTRA (hh, ss) and INTER (HH, SS) types. How intra and inter-type of salt bridges are populated in huPOP and pfPOP? To check this, we have presented Figure 2. Due to lower or absent frequency, we have compared only hh, HH, ss and SS populations between 5T88 and 3DDU. Several points are noteworthy from the figure. First, although both hh and ss are absent in isolated-core class, hh is present in isolated-surface class (Figure 2, f1-f2). Here, it is seen that intra-helical salt-bridges are much higher in 5T88 than 3DDU. Remarkably, in networked-core and networked-surface classes, although, 5T88 shows its presence with moderate to high frequency, it is almost absent in the case of 3DDU except for hh in isolated-core class (Figure 2, f3-f4). Third, in isolated-core and isolated-surface classes inter-helical (HH) salt-bridges are completely absent for both the proteins (Figure 2, g1-g2). However, in this case, inter-strand type (SS) shows its presence with much higher frequency for 5T88 (Figure 2, g1-g2). In networked-core and networked-surface population, both HH and SS are present. Interestingly, in these cases, 5T88 shows the higher relative population of these salt-bridges than 3DDU (Figure 2, g3-g4).

### Isolated and networked salt-bridges:

In this study, we have compared the salt bridge architecture of two proteins (3DDU and 5T88) that are functioning at two different environments. Salt-bridges are divided into two categories: isolated and networked. The details of these salt-bridges are secondary structure type (one of eleven possible combinations: HH, hh, SS, SS, HC, CH, SC, CS, HS, SH, CC), average distance, bondmultiplicity (one or more bonds between bridging partners), interresidue distances, core/surface locations (Co/Su), hydrogenbonded/ non-hydrogen bonded (HB/nHB), local/non-local (L/nL) and if local, its type. Table 3 and Table 4 show details of isolated and networked salt-bridges of 3DDU and 5T88 respectively. There are 19 and 28 isolated and networked salt-bridges in the case of 3DDU. The protein is 710 residues long. Although the length of the hyper-thermophilic protein (5T88) is shorter by about 100 residues, it has 27 and 39 isolated and networked salt-bridges. Surprisingly, although HIS mediated salt-bridges are frequent in both isolated and networked types of 3DDU, they are rare in the case of hyperthermophilic 5T88.

**Figure 3:**
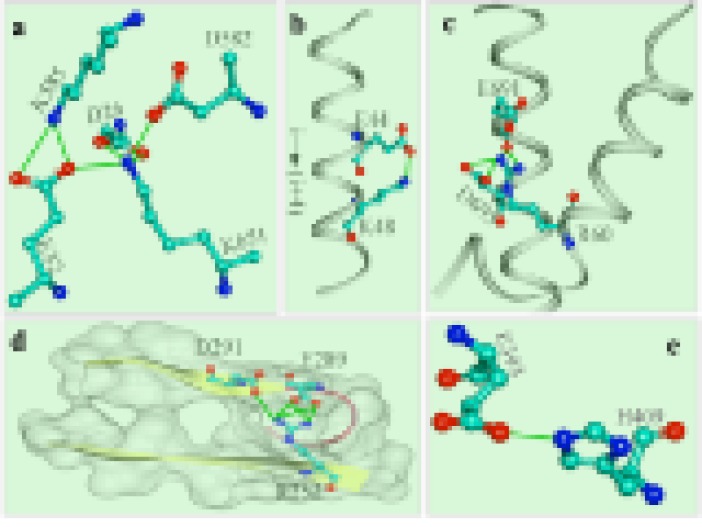
Representative salt-bridges from 3DDU and 5T88 such as multi-residue networked (a), INTRA-HELIX, i→ i+4 type (b), INTERHELIX and networked (c), INTER-STRAND and STRAND-COILED (d) and isolated (e) type salt-bridges.

Some typical salt-bridges are shown in Figure 3. A networked saltbridge (Figure 3a) is formed by more than one acidic and basic group. In the intra-helical salt-bridge, both the acidic and basic partners are seen to be present at the same side of the helix and further; the acidic-partner is present in the N-terminal end. The basic partner is present at (i+4) residues away, where (i) is the position of acidic partner. SBION2 [26], is the program that extracts this type of salt bridges from the crystal structure, which identifies this type as orientation-I (Figure 3b). In the inter-helix salt-bridge (Figure 3c), it is seen that basic partner is present in one helix and the acidic residues in the other. These three candidates together are forming a networked salt-bridge. A typical inter-strand salt bridge is shown in Figure 3d. It is present partly in the core and thus shown with is accessible surface. Here, base-partner is present in one strand and out of two acid-partners, one is present in the strand and the other is present in the coil. It is similar to the inter-helix salt bridge (Figure 3c) in terms of the arrangement. However, the three candidates together form a networked sat-bridge.

**Figure 4:**
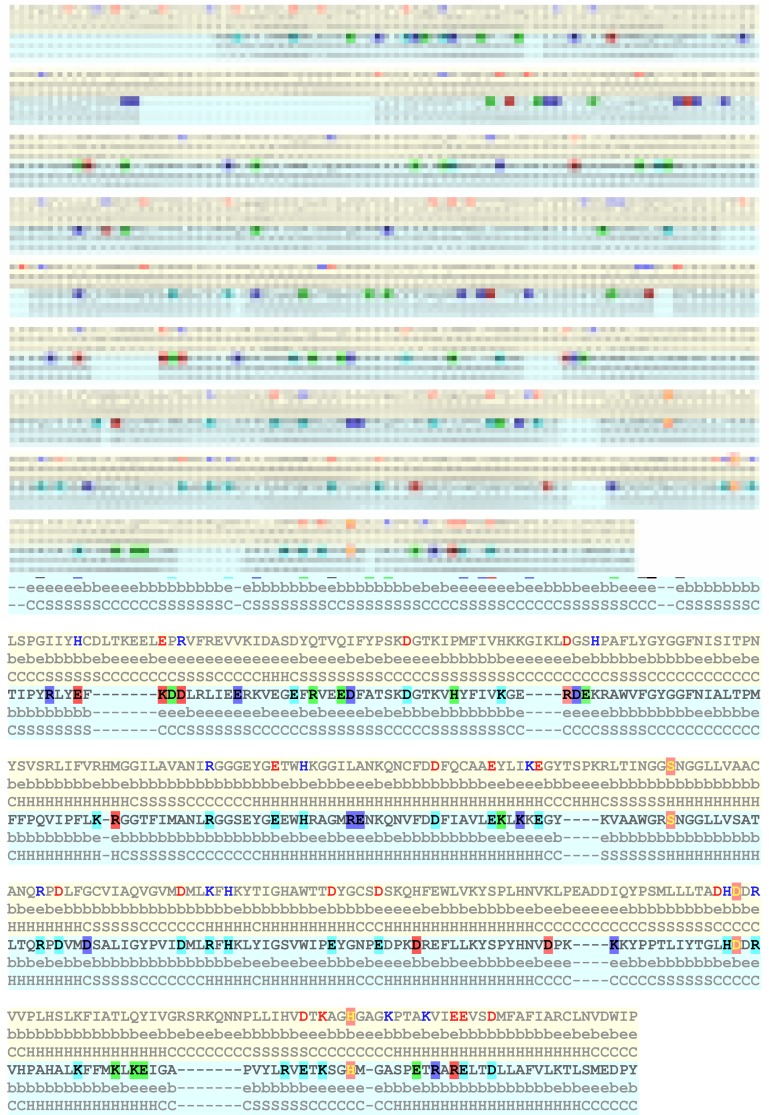
Details of salt-bridge residues in aligned sequences of 3DDU (upper) and 5T88 (lower). In the latter, salt-bridge residues are presented in different color shades. Green-shade: conservative substitution, blue-shade: non-conservative substitution, red: acid to base or base to acid substitution, cyan: unchanged with respect to 3DDU (upper). In 3DDU, salt bridge residues are shown in red (acidic) and blue (basic) colors. Core/surface (e/b) and helix/strand/coil (H/S/C) characteristics of each residue position are also shown in this alignment.

The alignment of 3DDU and 5T88 are shown in Figure 4 with details of salt-bridges and their core/surface and helix/strand/coil characteristics. Several points are noteworthy. First, although 5T88 has many deletions wrt 3DDU (Figure 4), its frequency of saltbridge residues is much higher than the latter (Table 3 and Table 4). At least four kinds of substitutions are notable in these salt-bridge forming residues. It is seen that salt bridge forming residue undergoes i) non-conservative, ii) conservative, iii) acid to base or base to acid types of substitutions wrt 3DDU. At the same time, about one-fourth of salt-bridge forming residues are kept positionally constant as 3DDU. Second, the active site residues (orange shade) and salt-bridge pattern remain largely similar in these two proteins. Third, the secondary structural positions and core-surface locations remain almost similar in both these proteins. Forth, PNCS, PCS and PU types each constitute 28% of partners of salt-bridge. Interestingly, 3/4 of each of the PNCS and PCS types are present in secondary structures. Rest 14% is constituted by PIC type.

## Discussion

### Substitutions and deletions are the major mechanisms of 5T88 over 3DDU:

Apart from mesophiles, organisms are also found under hyperthermophilic [1, 15], halophilic [2, 7, 8] and other hostile environments of the Earth. Due to very high temperature, thermophiles are living as a pure culture in their ecosystems as mesophiles can't grow there. Thermophilic proteins were shown to start functioning when the temperature of the medium is increased to the level of the growth temperature of these microbes [6]. These observations unequivocally suggest that these organisms and their biomolecules are adapted to their unusual ecosystems via evolution.

Pyrococcus furiosus is such a hyperthermophilic archaeon that thrive at the boiling point of water. As a consequence, the whole of its biochemical machineries are operating at this high-temperature. Because protein is the most exposed biomolecules in a cell for cellular functions and because high temperature (~80-100C) is also known to denature mesophilic proteins, understanding the stability of thermophilic proteins has been the major research focus for last 40 years [36]. Substitution, deletion, insertion, conjugation, and endosymbiosis are the mechanism of adaptation, of which thermophiles seem to relay more on substitution/deletion/insertion than conjugation/endosymbiosis for their adaptation, as a mix-culture state is the prerequisite for the success of the latter mechanism. Such a state is unlikely, as mesophiles can't withstand the ecological niche of thermophiles. Deletion is the preferred mechanism in thermophiles in general over the insertion, as the latter increase chain/loop flexibility and hampers overall packing of proteins at high temperature [36]. Thus, functionally identical proteins (orthologous) are shorter is the size in thermophiles than the mesophiles.

In our comparative analysis, we found 5T88, that introduced 13 deletions, is much shorter than 3DDU. Notably, these deletions are not always in the loop regions but also have overlap with secondary structural positions, may indicate this evolutionary decision is related to overcome the topological strain at high temperature. Remarkably, the hydrophobic residues in 5T88 are kept almost similar (little lower) than 3DDU. However, in the sequence of 5T88, both acidic and basic residues show their increase, of which higher and lower increases are constituted by surface and core of the protein respectively. What are the implications of maintenance of hydrophobic residues as 3DDU with the increase of acidic and basic ones in the surface and in the core of 5T88.

### The basis of thermostability in 5T88

The central theme of the study is to understand the evolutionary strategy that may have been designed in 5T88 in comparison to its mesophilic homologue for its stability and functionality under hyperthermophilic conditions. Keeping the level of normalized hydrophobic residues (in 5T88) as mesophilic one (3DDU) seems to be an evolutionary decision as at 100°C where pfPOP functions, the dielectric constant decrease to 55.51 [1]. In such a low dielectric medium, it appears that the formation of a typical mesophilic-likehydrophobic core is difficult under thermophilic conditions. The fact that hyperthermophilic situation trends to cause more flexibility, additional stabilizing interactions would be necessary to maintain the above mentioned characteristic balance. Intuitively, it appears that it is not the hydrophilic force that could replenish the deficit of required additional stability under hyperthermophilic conditions.

To understand the contribution of bound-waters (especially in core and cavity) to thermostability, we compared these interactions (isolated, bridged and a cavity in core and surface) between 5T88 and 3DDU. We observed that such interactions between boundwaters and atoms of 5T88 are much less than 3DDU. We found the normalized frequency of salt-bridges is much higher in 5T88 than 3DDU. Similar observations are also entertained in many thermophilic proteins [1, 10, 11, 15]. We further partitioned the overall increase of salt-bridges into isolated and networked categories, and core and surface classes. Salt-bridges of each class is further partitioned into different binary items such as single vs multiple bonded, local vs non-local, hydrogen bonded vs nonhydrogen bonded, in secondary-structure vs in coiled-structure, intra-helix vs intra-strand and inter-helix vs inter-strand using automated procedure [25, 26]. Comparison of each binary item of salt-bridges between 5T88 and 3DDU allows us to reach to the conclusion that it is salt-bridges but not the water-protein and hydrophobic interactions that act as the prime force for replenishing the deficit of required additional stability in the former. The increase of salt-bridges at all level also accounts for the higher melting temperature of these proteins. It is similar to the Tm of DNA segment, where it is always less in AT-rich DNA than a GC-rich one. The increase of salt-bridge interactions at all level of binary items may account the enhanced stability and the Tm of thermophilic protein, 5T88 in particular and others in general.

### Evolutionary design of salt-bridges in 5T88:

Earlier we pointed out partners (acidic and basis residues) of saltbridges increases both in the core and in the surface. We also pointed out the difference in homologous positions of 5T88 (hyperthermophilic) from 3DDU (mesophilic) is 75%. What are the types of substitutions in this positional difference in terms of saltbridge partners? In 5T88, 109 partners are involved in forming 27 isolated and 39 networked salt-bridges. From the alignment, we identified and classified these 109 partners into 4 classes such as partners i) remain positionally conserved (PU), ii) undergo nonconservative substitutions (PNCS), iii) undergo conservative substitutions (PCS) and iv) inter-changed from acidic to basic or vice versa (PIC). In PU, PNCS, PCS and PIC groups there are 31 (28%), 31 (28%), 31 (28%) and 16 (15%) partners. In PNCS and PCS, 23 (74%) and 22 (71%) are present in the secondary structures (Helices and Strands). It has been claimed that NCS has little or no structural role in the proteins [37], which contradict with our observations. The appearance of NCS in the secondary structure seems to be related with the tuning of the topology of 5T88 [38]. PIC class seems to be critical in changing the mesophilic design of salt-bridges into a thermophilic one. Active site and other binding site, salt-bridges are maintained by PC class and the PCS class allows increasing the proportion of salt-bridges (71% in secondary structure) by keeping the overall properties of the protein similar. Overall, these four classes of salt-bridge partners play a critical role in increasing the frequency (PCS), in producing new design (PNCS), in the maintaining (PC) and abolishing mesophilic pattern (PIC) of salt-bridges.

## Conclusion

We performed a comprehensive analysis of salt-bridges in hyper thermophilic prolyl oligo-peptidase (PDB ID: 5T88) in comparison to its mesophilic homologue (PDB ID: 3DDU). Majority of increased acidic and basic residues in the core and in the surface form additional isolated (core and surface) and networked (core and surface) salt-bridges. It is found that 5T88 has more normalized frequency than that of 3DDU. These enhanced levels of salt-bridges have relation with the thermo stability and higher Tm of the protein. It is further found that 30% of partners of salt bridges are for maintenance as mesophiles for the active site and other sites. Moreover, 28% of partners in salt-bridges are due to NCS and 75% of which are in the secondary structures. This population of saltbridges is important for the topology of the protein in hyper thermophilic conditions. The remaining 14% of the partners of saltbridges are inter-changed types (e.g. acid to the base and vice versa). Overall, the comparative study on salt-bridges provides insights into the thermostability, which have potential implication in protein-engineering.

## Conflict of Interest

Authors declare no conflict of interest

## Figures and Tables

**Table 1:** Database details of huPOP (3DDU) and pfPOP (5T88).

Items	Mesophilic	Thermophilic
Organism	Homo sapiens	Pyrococcus furiosus
Protein	Prolyl oligopeptidase (EC:3.4.21.26)	Prolyl oligopeptidase (EC:3.4.21.26)
Length	710	616
UniProt ID	P48147	Q51714
RCSB ID	3DDU	5T88
Resolution	1.56 Å	1.9 Å
Chains in str.	One (monomer)	two (dimer)
Shell-waters	A: 1250	A: 461; B:487
HELIX (DSSP)	22% helical (22H; 161R)	24% helical (18H; 150R)
SHEEL (DSSP)	32% β-sheet (39S; 230R)	39% β-sheet (36S; 242R)
Str. Structure; H helices; S strands; R amino acid residues; DSSP Dictionary of Secondary Structure of Proteins [21]		

**Table 2:** Comparative analysis of sequence and structural properties of huPOP (3DDU) and pfPOP (5T88)

Items	3ddu		5t88
amino acids in seq.	710		616
hydrophobic	49%		48.30%
hydrophilic	51%		51.70%
Acidic and Basic	12.2% and 13.2%		16.2% and 15.5%
Aliphatic Index	82.8		84.9
pI	6.14		5.63
GRAVY	-0.23		-0.39
NCS:CS substitutions	0.45		0.51
Sequence difference (%)	-		74.9
Surface comp.	HB=11.2%; HL=31.5%; a+b=18.7%		HB 9.0%; HL=33.0%; a+b=18.2%
Core comp.	HB=32%; HL=25.2%; a+b=7.8%		HB=34.5%; HL=23.5%; a+b=8.3%
Water-protein	Total water	1250 moles	546 moles
interactions	Total int. (=≤3.2Å)	160/100 residues	75/100 residues
	Isolated int.	95/100 residues	66/100 residues
	Bridge (by prot.) Int.	41/100 residues	5/100 residues
	Bridge (by wat.) Int.	24/100 residues	6/100 residues
	Core int.	70 (C:H:S=33:22:15)	33 (C:H:S =14:09:10)
	Surface int.	90 (C:H:S =50:21:22)	42 (C:H:S =21:10:11)
	Internal cavity	31	29
Seq. sequence; Comp. composition; HB hydrophobic; HL hydrophilic; a+b acidic+basic; NCS non-conservative; CS conservative; int. interaction; prot. Protein; wat. Water; C coil; H helic; S strand			

**Table 3:** Isolated and networked salt-bridges of 3DDU (A chain) along with details on different binary items. SST secondary structure type; Mu Multiplicity; IRD Inter residue distance; co core; su surface; HB hydrogen bonded; L local; nL non-local; LDT Local distance type; ISB isolated SB; NSB network SB.

ISB 19 NSB 28 (3ddu_A)	SST	Av. Dist.	Mu	IRD	B vs E	H vs nH	L vs nL	LDT
K677-D122	CC	3.2	2	556	E	H	nL	.
H466-D463	CC	3.7	1	4	E	nH	L	3
K196-D35	CC	3.4	2	162	E	H	nL	.
H640-D639	CC	2.8	1	2	E	H	L	1
R11-E13	CC	3.4	4	3	E	H	L	2
H680-D641	CC	3	2	40	B	H	nL	.
K539-E540	hh	3.5	2	2	E	H	L	1
K48-E44	hh	3.3	1	5	E	H	L	4
K23-D18	SS	3.3	1	6	E	H	L	.
H409-E393	SS	2.7	1	17	B	H	nL	.
H587-D603	HC	3.6	1	17	E	nH	nL	.
K325-D320	HC	3.4	2	6	E	H	L	5
K684-E692	CH	2.6	1	9	B	H	L	8
R245-D265	CS	3.3	4	21	E	H	nL	.
K389-E296	CS	3.3	2	94	E	H	nL	.
R420-E418	SC	3	2	3	E	H	L	2
R85-E137	SC	3.5	3	53	B	nH	nL	.
R260-D284	SC	3.4	3	25	E	H	nL	.
K688-D675	HS	3.1	2	14	E	H	nL	.
H355-D336	SC	3	2	20	E	H	nL	.
H355-D356	SC	3.8	1	2	E	nH	L	1
R306-E287	CC	3.3	2	20	E	H	nL	.
R306-E323	CH	3.3	4	18	E	H	nL	.
H213-D166	SS	2.7	1	48	B	H	nL	.
H213-D222	SH	3.1	2	10	B	H	nL	.
K651-D26	HC	3	2	626	B	H	nL	.
K651-E32	HH	2.8	1	620	B	H	nL	.
K651-D582	HC	2.9	1	70	B	H	nL	.
R60-E691	HH	3.2	2	632	E	H	nL	.
R60-D695	HH	2.9	2	636	B	H	nL	.
R505-D529	CH	3.3	4	25	B	H	nL	.
R505-D446	CC	3.4	4	60	E	H	nL	.
K585-E32	HH	3.4	2	554	B	H	nL	.
K585-D26	HC	2.8	1	560	B	H	nL	.
R252-D291	SS	3.7	1	40	E	nH	nL	.
R252-E289	SC	3.2	4	38	B	H	nL	.
H515-E512	hh	3	2	4	B	H	L	3
H515-D598	HH	3.3	2	84	B	H	nL	.
R567-E535	HH	3.5	2	33	E	H	nL	.
R567-D569	HH	3.3	4	3	E	H	L	.
K390-E134	CC	2.9	1	257	E	H	nL	.
H180-E134	CC	3.7	3	47	E	nH	nL	.
H307-E323	CH	3.5	1	17	E	H	nL	.
K64-E691	HH	3.3	2	628	E	H	nL	.
K303-D291	SS	3.5	1	13	B	H	nL	.
R643-D149	CC	3.3	4	495	B	H	nL	.
R128-D149	SC	3.6	2	22	B	nH	nL	.

**Table 4:** Isolated and networked salt-bridges of 5T88 along with details on different binary items is given. SST secondary structure type; Mu Multiplicity; IRD Inter residue distance; co core; su surface; HB hydrogen bonded; L local; nL non-local; LDT Local distance type; ISB isolated SB; NSB network SB.

ISB 27 NSB 39 (5T88_B)	SST	Av. Dist	Mu	IRD	B vs E	H vs nH	L vs nL	LDT
R391-E393	CC	3.1	2	3	E	H	L	2
K204-D202	CC	3.5	1	3	E	nH	L	2
K589-E94	CC	2.6	2	496	E	H	nL	.
R158-D119	CC	3.7	2	40	E	nH	nL	.
K547-D544	CC	2.5	1	4	E	H	L	3
K574-E577	hh	3.6	1	4	E	nH	L	3
K37-E33	hh	2.7	1	5	E	H	L	4
K85-E88	hh	3	2	4	E	H	L	3
K25-E29	hh	2.6	1	5	E	H	L	4
R602-E598	hh	3.3	2	5	E	H	L	4
R15-D3	hh	3.3	4	3	E	H	L	2
K232-E218	SS	3.9	1	15	E	nH	nL	.
R55-D329	SS	3.7	1	275	B	nH	nL	.
R155-E181	SS	3.4	4	27	E	H	nL	.
H383-E371	SS	2.5	1	13	B	H	nL	.
K262-E273	SS	3.3	1	12	E	H	nL	.
R296-E305	SS	3.3	4	10	B	H	nL	.
K388-D392	SC	3.6	2	5	E	nH	L	4
K199-E104	SC	3.2	2	96	E	H	nL	.
K315-D354	SC	2.7	2	40	E	H	nL	.
K108-D103	SC	2.9	1	6	B	H	L	5
R172-D164	SC	3.1	4	9	B	H	L	8
R368-E366	SC	3	5	3	E	H	L	2
R182-E179	SC	3.5	1	4	E	nH	L	3
K465-D495	HC	2.8	2	31	E	H	nL	.
H510-E526	HC	3	1	17	E	H	nL	.
K255-E283	HS	2.7	1	29	E	H	nL	.
K352-E350	CS	3.6	1	3	B	nH	L	2
K352-E333	CC	3.4	1	20	E	H	nL	.
R585-E587	ss	3.5	2	3	B	H	L	2
R585-E603	SH	3.9	1	19	B	nH	nL	.
R585-D606	SH	2.9	2	22	B	H	nL	.
K570-D505	HC	2.8	1	66	B	H	nL	.
K570-E9	HH	2.9	1	562	B	H	nL	.
K570-D3	HC	2.8	1	568	B	H	nL	.
R447-E448	Hh	3.6	2	2	E	nH	L	1
R447-D530	HH	3.4	4	84	B	H	nL	.
R347-E360	SS	2.9	2	14	E	H	nL	.
R347-D320	SC	3.2	4	28	E	H	nL	.
R432-D456	CH	3.4	4	25	B	H	nL	.
R432-D378	CC	3.3	4	55	B	H	nL	.
R124-E136	SS	3.1	2	13	E	H	nL	.
R124-E90	SH	3.3	4	35	E	H	nL	.
K18-E22	Hh	3.9	1	5	E	nH	L	4
K18-E21	Hh	3.8	1	4	E	nH	L	3
K463-E467	Hh	2.8	1	5	E	H	L	4
K463-D372	HS	3.5	1	92	E	H	nL	.
H442-E521	HH	3	2	80	B	H	nL	.
H442-E439	Hh	2.9	2	4	B	H	L	3
R490-D492	HH	3.3	4	3	E	H	L	.
R490-E462	HH	3	1	29	E	H	nL	.
R70-D83	SS	3.6	4	14	B	nH	nL	.
R70-D69	SC	3.7	2	2	E	nH	L	1
R334-E350	CS	3.2	2	17	B	H	nL	.
R600-E603	Hh	3	1	4	B	H	L	3
R26-E23	Hh	2.9	1	4	B	H	L	3
K576-E23	HH	3.3	1	554	E	H	nL	.
K313-E316	ss	3.1	1	4	E	H	L	3
K303-E316	SS	3.7	1	14	E	nH	nL	.
R508-E9	HH	3.1	4	500	B	H	nL	.
K138-E120	CC	3.5	1	19	E	H	nL	.
K159-E120	CC	3.9	1	40	E	nH	nL	.
K291-D353	SC	3.9	1	63	E	nH	nL	.
R289-D353	CC	3.5	4	65	E	H	nL	.
K421-E41	HH	3.5	2	381	E	H	nL	.
R422-E41	HH	3.1	4	382	E	H	nL	.
